# Supplementary material for: Recognizing affiliation in colaughter and cospeech
Source: R Soc Open Sci. 2020 Oct 7;7(10):201092. doi: 10.1098/rsos.201092 (PMC7657881; doi:10.1098/rsos.201092)
Supplement: Supplementary Materials [file rsos201092supp1.doc]

**SUPPLEMENTARY MATERIAL**

Recognizing affiliation in colaughter and cospeech

Gregory A. Bryant, Christine S. Wang, & Riccardo Fusaroli

1. Supplementary Signal Detection Analysis Details
2. Analysis of the affiliation ratings
3. Full text of experimental instructions
4. Laughter examples
5. Cospeech transcriptions

**1. Supplementary Signal Detection Analysis Details**

1.1. Model comparison

Table S1: Model comparison including all experimental manipulations

| **Model** | **Looic** | **SE of Looic** | **Difference from best model** | **SE of the difference** | **Weights** |
| --- | --- | --- | --- | --- | --- |
| Model 4 | 12960.81 | 69.48 | NA | NA | 0.83 |
| Model 2 | 12995.80 | 68.34 | 34.99 | 14.40 | 0.17 |
| Model 3 | 13445.41 | 58.84 | 484.60 | 42.90 | 0 |
| Model 1 | 13485.50 | 57.36 | 524.70 | 44.69 | 0 |
| Model 0 | 13810.38 | 46.12 | 849.58 | 55.61 | 0 |

1.2. Individual variability in effects

Besides the general (i.e., population-level) effects, we also examined individual variability in effects by participant to better assess the robustness of the findings. In other words, we explored how the effects reported above worked within participants.

Not all participants were able to better discriminate colaughter than cospeech. As can be seen in Figure 5, there was variability in the effect, with only 56 participants out of 108 clearly displaying an effect (population level effect of 0.37, average expected individual variability 0.33).

Figure S1. Average expected sensitivity for original stimuli per each participant. Each line represents a different participant shift in sensitivity to original colaughter vs. original cospeech.


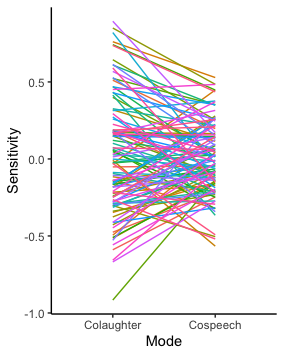


Similarly, not all participants showed analogous effects of the speed manipulation. As can be seen in Figure S2, there was substantial variability, with only 58 participants out of 108 clearly displaying an effect (population level effect of 0.19, average expected individual variability 0.13).

Figure S2. Average expected criterion (false positives rate) for original and sped-up stimuli per each participant. Each line represents a different participant shift in tendency to respond “friends” to original stimuli produced by strangers vs. the sped-up version of the same stimuli.


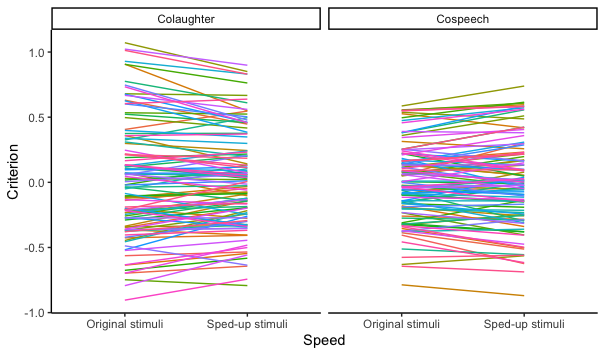


These exploratory analyses indicate that while the population-level pattern seems robust, there is reasonably large individual variability in how sensitive participants are to the different conditions. Further exploration of individual variability is presented in Table S2 and Figure S3, and of stimulus variability in Figure S4.

Table S2: Standard deviations of individual variability within each condition. Note how the standard deviation was similar across conditions, with large overlapping across credibility intervals:

____________________________________________________________________________

Non-Familiar colaughter (original speed): 0.34 (95% CIs 0.25 0.45)

Familiar colaughter (original speed): 0.26 (95% CIs 0.17 0.36)

Non-Familiar colaughter (sped-up): 0.36 (95% CIs 0.27 0.47)

Familiar colaughter (sped-up): 0.35 (95% CIs 0.26 0.46)

Non-Familiar cospeech (original speed): 0.48 (95% CIs 0.38 0.60)

Familiar cospeech (original speed): 0.42 (95% CIs 0.31 0.53)

Non-Familiar cospeech (sped-up): 0.42 (95% CIs 0.32 0.53)

Familiar cospeech (sped-up): 0.48 (95% CIs 0.37 0.62)

____________________________________________________________________________

Figure S3. Average expected rate of “friends” response by condition per each participant. Each line represents a different participant shift in response to stimuli produced by friends vs. those produced by strangers. The population-level patterns are largely conserved across most participants, but less so in cospeech.


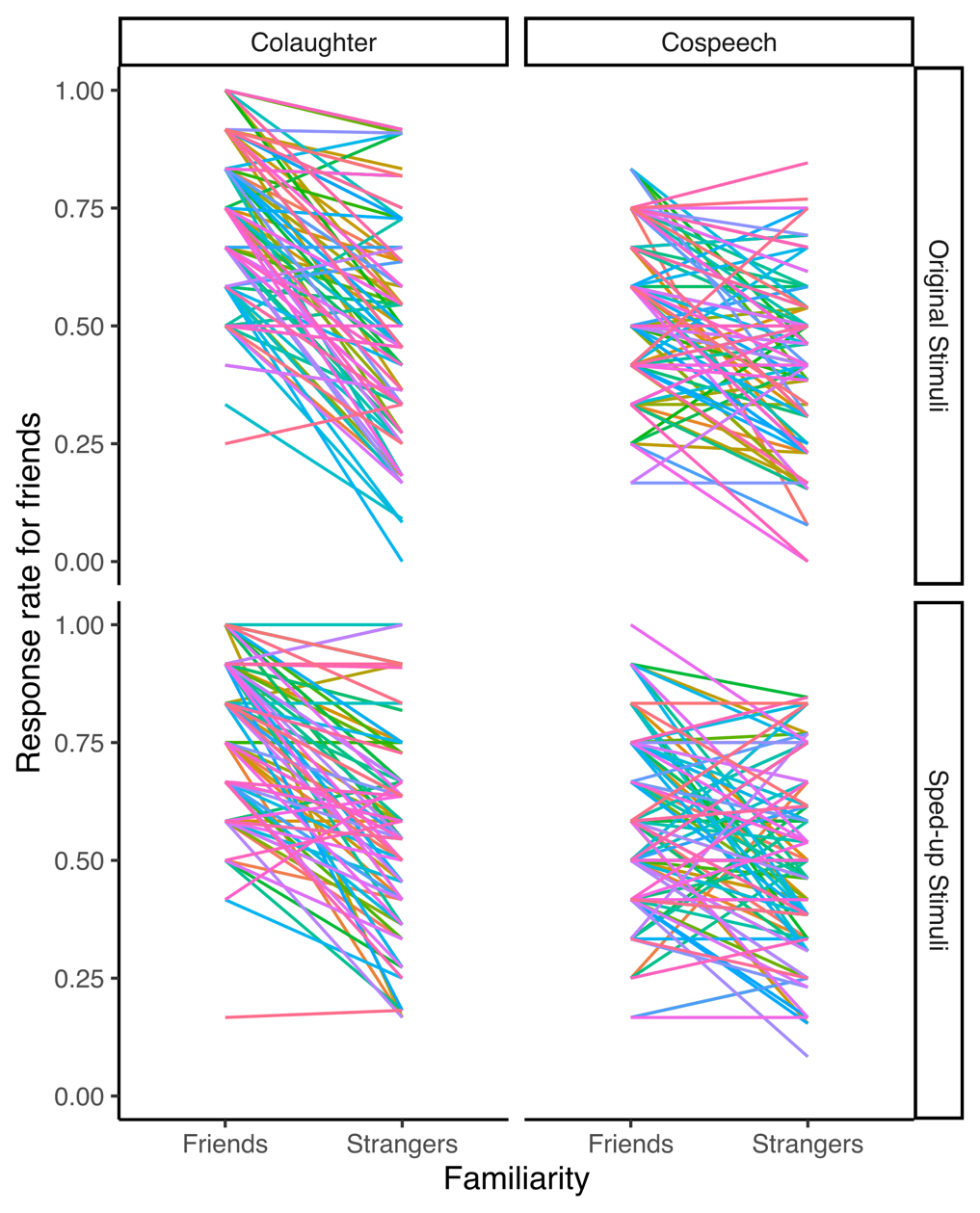


Figure S4. Distributions of the average expected rate of “friends” response by stimulus in each condition. We first calculated the average rate per each stimulus, then represented the distribution of these rates within each condition. We overlayed colaughter and cospeech by each condition to see whether the variance of the distribution was qualitatively different, which would indicate different heterogeneity of the stimuli by condition. The plot highlights how laughter in friends is a clear signal across the stimuli.


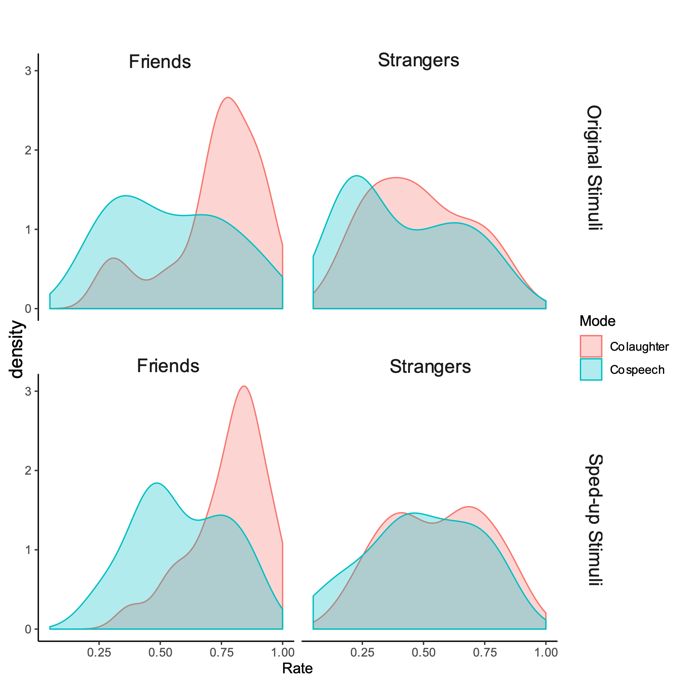


**2. Analysis of the affiliation ratings**

In the experiment, participants were asked to judge whether they thought the pair of speakers were friends or strangers (Question 1), and then they were asked “How much do you think these people liked each other?” The response went from 1 being “Not at all” to 7 being “very much.” Figure S5 represents the raw data by condition.

In order to assess whether the patterns observed on the “friends” judgments (Question 1) would hold on the ordinal ratings, these data were analyzed using a Bayesian multilevel ordinal regression model with a cumulative logit link, following procedures analogous to those reported for the main analysis in the manuscript (predictive checks, model quality checks, etc). Ratings were entered as ordinal outcome; familiarity, type of stimulus (colaughter vs. cospeech), and speed (original vs. sped-up) as interacting predictors, potentially varying by participants (random slopes over participant). Ratings were also modeled as potentially varying by specific stimulus (random intercept of stimulus). We chose as priors normal distributions centered at 0 and with a standard deviation of 0.3 for intercepts and effects of each condition, as well as positively truncated normal distributions centered at 0 with a standard deviation of 0.1 for the varying effects.

As expected, participants rated colaughter produced by friends (original stimuli) higher than that produced by strangers (effect of familiarity as an average increase in chance to have a higher rating on a log-odds scale: 0.86, 95% CIs 0.55 1.16, 100% credibility). This effect was more pronounced for colaughter than it was for cospeech (difference in effect between colaughter and cospeech: 0.58, 95% CIs 0.14 1.03, 99% credibility).

Also as predicted, speeding up colaughter induced ratings of higher affiliation across stimuli produced by friends and strangers (0.13, 95% CIs 0.04 0.22, 99% credibility), but the effect was driven by stimuli produced by strangers (0.28, 95% CIs 0.15 0.4, 100% credibility vs no effect in those produced by friends: -0.01, 95% CIs -0.14 0.12, 44% credibility). As in the main analysis, the effects of increased speed was not credibly different between colaughter and cospeech (0.03, 95% CIs -0.1 0.16, 66% credibility).

Figure S3. Ratings for Question 2: “How much do you think these people liked each other?”


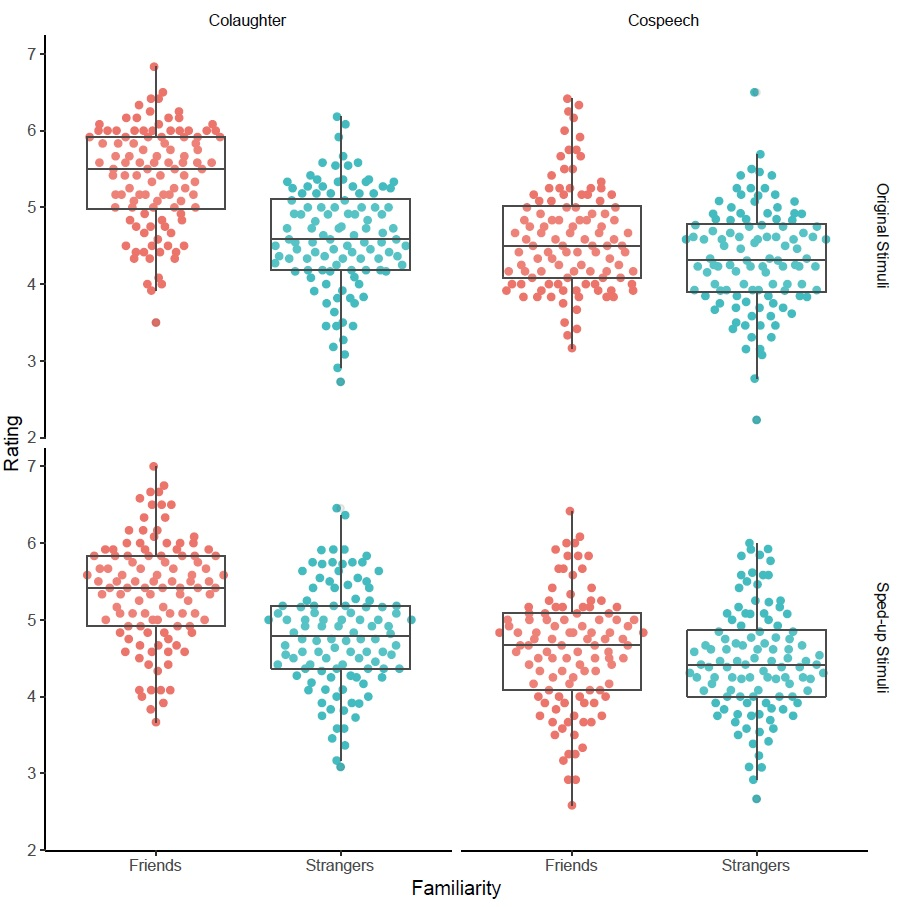


**3. Full text of experimental instructions**

In this experiment we will have you listen to recordings of people, in pairs, laughing or talking together in a conversation, and then answer two questions about each recording.

Some of the pairs of people were friends at the time of the recording, and others were complete strangers who were meeting for the first time. We will ask you whether you think the people interacting were friends or were strangers, and then we will ask you how well the people liked each other.

The recordings of the people laughing or talking are very short. Before we begin with the actual study, you will be able to practice with one recording so that you will become familiar with the procedure. Press the space bar when you are ready to begin the practice session.

**4. Stimulus examples of colaughter**

Friends, Colaughter (friends_colaughter.wav)

Friends Cospeech (friends_cospeech.wav)

Strangers Colaughter (strangers_colaughter.wav)

Stranger Cospeech (stranger_cospeech.wav)

Friends Colaughter Sped-up (friends_colaughter_fast.wav)

Strangers Colaughter Sped-up (strangers_colaughter_fast.wav)

Friend Cospeech Sped-up (friends_cospeech_fast.wav)

**5. Cospeech transcriptions**

Table S3. Complete text of cospeech stimuli transcriptions

| Stimulus ID | Speaker | Utterance |
| --- | --- | --- |
| Friends 2-1 | 1 | Yeah |
|  | 2 | I don’t know |
| Friends 2-2 | 1 | You know what I mean? |
|  | 2 | Yeah |
| Friends 3-1 | 1 | Just him or whatever |
|  | 2 | Yeah |
| Friends 3-2 | 1 | Fit in there like |
|  | 2 | It’s more |
| Friends 4-1 | 1 | Yeah, it’s, it’s, I don’t, I don’t even |
|  | 2 | You’re a, you’re a woman |
| Friends 4-2 | 1 | I don’t know |
|  | 2 | That…that communication |
| Friends 8-1 | 1 | But like, I understand |
|  | 2 | That’s pretty whack |
| Friends 8-2 | 1 | But like |
|  | 2 | Yeah ‘cause it goes up |
| Friends 27-1 | 1 | And three |
|  | 2 | Yeah |
| Friends 27-2 | 1 | It’s kind of fun |
|  | 2 | Yeah |
| Friends 28-1 | 1 | All the time |
|  | 2 | Yeah |
| Friends 28-2 | 1 | You know, like |
|  | 2 | Yeah |
| Friends 29-1 | 1 | So I have really good speakers |
|  | 2 | Mmhmm |
| Friends 29-1 | 1 | Forgot |
|  | 2 | In the navy |
| Friends 30-1 | 1 | Yeah so |
|  | 2 | Oh really? |
| Friends 30-2 | 1 | Why? |
|  | 2 | Bounce |
| Friends 31-1 | 1 | Oh… |
|  | 2 | And uhh |
| Friends 31-2 | 1 | Yeah |
|  | 2 | Relationship all the time |
| Friends 34-1 | 1 | How sad |
|  | 2 | And she’d call |
| Friends 34-2 | 1 | That’s crazy |
|  | 2 | Yeah |
| Friends 40-1 | 1 | Umm |
|  | 2 | What, what’s that? |
| Friends 40-2 | 1 | Yeah dude |
|  | 2 | If you’re bored |
| Friends 41-1 | 1 | Oh god |
|  | 2 | And like |
| Friends 41-2 | 1 | Yeah |
|  | 2 | Such a good idea |
| Strangers 11-1 | 1 | Definitely |
|  | 2 | That’s good |
| Strangers 11-2 | 1 | Yeah |
|  | 2 | Was it really? Okay |
| Strangers 12-1 | 1 | Stuff like that |
|  | 2 | Oh, that’s lame |
| Strangers 12-2 | 1 | Are you serious? |
|  | 2 | Yeah |
| Strangers 14-1 | 1 | Every…like yeah |
|  | 2 | Really? |
| Strangers 14-2 | 1 | Come down from some party |
|  | 2 | Oh yeah |
| Strangers 17-1 | 1 | And then all of a sudden |
|  | 2 | Mmhmm |
| Strangers 17-2 | 1 | People around |
|  | 2 | Well |
| Strangers 18-1 | 1 | But never got one |
|  | 2 | Yeah |
| Strangers 18-2 | 1 | Err, something like that |
|  | 2 | Go for longer |
| Strangers 19-1 | 1 | You know what I mean? |
|  | 2 | Yeah |
| Strangers 19-2 | 1 | Yeah, I don’t like it either |
|  | 2 | Like, like car eats up gas |
| Strangers 20-1 | 1 | You know, I don’t know |
|  | 2 | Yeah |
| Strangers 20-2 | 1 | Played basketball |
|  | 2 | Yeah |
| Strangers 21-1 | 1 | Yeah |
|  | 2 | Talk about |
| Strangers 21-2 | 1 | Mmhmm |
|  | 2 | And she like |
| Strangers 24-1 | 1 | Did you |
|  | 2 | And uhh |
| Strangers 24-2 | 1 | Anymore, and then |
|  | 2 | Mmhmm |
| Strangers 26-1 | 1 | But it’s like opposite |
|  | 2 | Oh |
| Strangers 26-2 | 1 | It’s not that far |
|  | 2 | That makes sense |
| Strangers 35-1 | 1 | Like in the hallway |
|  | 2 | Uh huh |
| Strangers 35-2 | 1 | Seems like it will |
|  | 2 | Oh well |
| Strangers 36-1 | 1 | Yeah that is really |
|  | 2 | It’s weak |
| Strangers 36-2 | 1 | So |
|  | 2 | That sucks |

*Note:* Conversation numbers are consistent with previous publications using these stimuli.
